# Inhibitory effects of the nanoscale lysate derived from xenogenic dental pulp stem cells in lung cancer models

**DOI:** 10.1186/s12951-023-02218-1

**Published:** 2023-12-18

**Authors:** Yan He, Ruohan Li, Wenting She, Yilong Ai, Kesheng Li, Tushar Kumeria, Ziran Jiang, Qing Shao, Chen Zou, Abdullkhaleg Ali Albashari, Xingxiang Duan, Qingsong Ye

**Affiliations:** 1 Center of Regenerative Medicine, Renmin Hospital of Wuhan University, Wuhan, 460030 Hubei China; 2 Institute for Regenerative and Translational Research, Tianyou Hospital of Wuhan University of Science and Technology, Wuhan, 460030 Hubei China; 3 Foshan Stomatological Hospital, School of Medicine, Foshan University, Foshan, 528000 Guangdong China; 4 School of Materials Science and Engineering, University of New South Wales, Kensington, Sydney, NSW 2052 Australia; 5 School and Hospital of Stomatology, Wenzhou Medical University, Wenzhou, 324025 Zhejiang China; 6 Department of Plastic Surgery, Renmin Hospital of Wuhan University, Wuhan, 460030 Hubei China; 7 Department of Stomatology, Renmin Hospital of Wuhan University, Wuhan, 460030 Hubei China; 8 Department of Oral and Maxillofacial Surgery, Massachusetts General Hospital, Harvard Medical School, Boston, MA 02114 USA

**Keywords:** Dental pulp stem cells lysate, Nanoscale, Lung cancer, Proliferation, Metastasis

## Abstract

**Background:**

Lung cancer is a highly prevalent malignancy and has the highest mortality rate among all tumors due to lymph node metastasis. Bone marrow and umbilical cord-derived mesenchymal stem cells (MSCs) have demonstrated tumor-suppressive effects on lung cancer. This study investigated the effects of DPSC lysate on proliferation, apoptosis, migration and invasion of cancer cells were studied in vivo and in vitro.

**Methods:**

The proliferation, apoptosis, and migration/metastasis were evaluated by cell counting kit-8 assay, Annexin-V and propidium iodide staining, and the transwell assay, respectively. The expression levels of apoptosis-, cell cycle-, migration-, and adhesion-related mRNA and proteins were measured by qRT-PCR and western blot. The level and mRNA expression of tumor markers carcino embryonic antigen (CEA), neuron-specific enolase (NSE), and squamous cell carcinoma (SCC) were measured by Enzyme-linked immunosorbent assay (ELISA) and qRT-PCR. Finally, a tumor-bearing mouse model was constructed to observe the tumor-suppressive effect of DPSC lysate after intraperitoneal injection.

**Results:**

DPSC lysate decreased the viability of A549 cells and induced apoptosis in lung cancer cells. Western blot confirmed that levels of Caspase-3, Bax, and Bad were increased, and Bcl-2 protein levels were decreased in A549 cells treated with DPSC lysate. In addition, DPSC lysate inhibited the migration and invasion of A549 cells; downregulated key genes of the cell cycle, migration, and adhesion; and significantly suppressed tumor markers. Xenograft results showed that DPSC lysate inhibited tumor growth and reduced tumor weight.

**Conclusions:**

DPSC lysate inhibited proliferation, invasion, and metastasis; promoted apoptosis in lung cancer cells; and suppressed tumor growth- potentially providing a cell-based alternative therapy for lung cancer treatment.

**Graphical Abstract:**

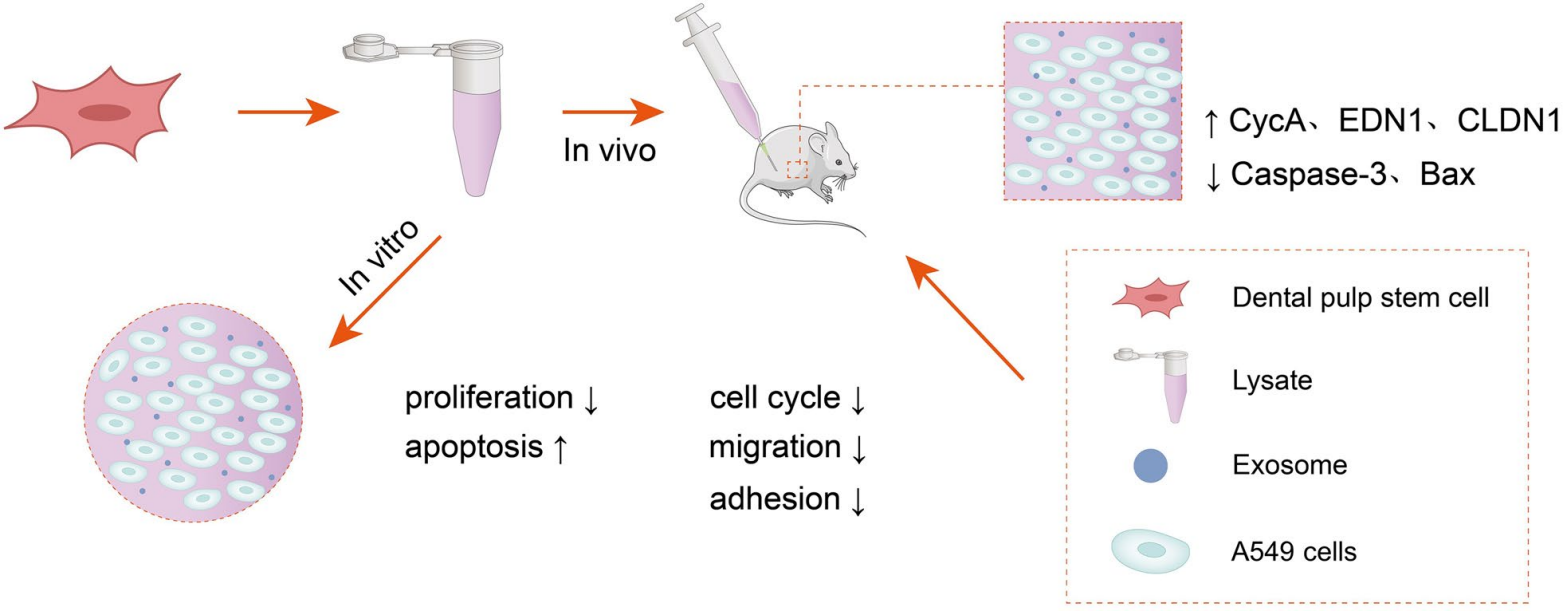

## Introduction

Lung cancer is the leading cause of cancer death (especially for men over 40 and women over 60) [1] with an estimated 2 million new cases and 1.76 million deaths each year [2]. Despite the current advances in diagnosis and treatment, the main treatments for lung cancer are surgery, radiotherapy, and chemotherapy; and the 5-year survival rate remains below 20% [3]. Consequently, the development of effective methods for treating lung cancer is imperative.

Mesenchymal stem cells (MSCs) are a class of pluripotent stem cells with multifunctional differentiation potential that can be isolated from bone marrow, adipose, placenta, umbilical cord and dental pulp [4]. MSCs are capable of self-renewal and differentiation into osteoblasts, chondrocytes, and adipocytes. In addition, MSCs play important paracrine roles and are extensively involved in immune regulation, cell proliferation, apoptosis, endogenous precursor cell regeneration, vascular regeneration, and release of growth factors to modify the endothelial or epithelial cell injury response [5–8]. Meanwhile, MSCs are an important component of the tumor microenvironment [9], and may differentiate at tumor sites [10]. As plastic cells, MSCs determine their phenotype and function according to the cytokines of neighboring tumor-infiltrating immune cells. Depending on the tumor microenvironment to which they are exposed, MSCs can acquire both pro- and anti-tumor phenotypes, thereby enhancing or inhibiting tumor growth [11]. Therefore, even if MSCs of the same origin were used to treat different tumor cells, the resulting effects were not the same [12]. On the other hand, due to the natural property of MSCs to be attracted by multiple chemokines released from tumor tissues or tumor microenvironment and target homing to tumor tissues, MSCs and their exosomes can be used as carriers to deliver antitumor drugs in tumor cells, reducing tumor viability and aggressiveness [13, 14]. As research continued, MSCs were found to not only serve as carriers carrying tumor-killing genes, but also to have tumor suppressive effects themselves [15–18].

Dental pulp stem cells (DPSC) are isolated from the third molars and possess all the properties of MSCs. The third molars are usually discarded as medical waste when they are no longer needed and are an abundant and ethically uncontroversial source of stem cells [19]. Therefore, dental pulp may be a valuable non-invasive alternative source of MSCs [20]. Cell-free therapy is an attractive emerging therapeutic technique in stem cell applications [21], excluding some of the limitations and risks associated with cell therapy [22, 23], such as avoiding blockage of the microvascular system and transformation into inappropriate cell types [24, 25]. In addition, cell-free therapies demonstrate significant advantages in terms of production, storage, and standardization [26]. Exosomes are typical examples of acellular therapy, but the low yield and difficulty in extraction severely limit the clinical application of exosomes [27]. Previous studies revealed that MSCs could suppress tumor progression in different cancers by paracrine signaling via MSC-derived extracellular vesicles [28]. Cell lysate is simple to produce, Human platelet lysate has been widely used in many fields, but there are few researches on stem cell lysate. Some studies have reported that stem cell lysate has a good effect on colitis and photoaging models [19, 29], but there is no relevant study on the efficacy of tumor. In this study, we assessed whether DPSC lysate was a cost-effective treatment strategy and if it has an inhibitory effect on lung cancer cells.

## Methods

### Cell culture and DPSC characterization

Five human third molars samples collected from five donors (16–30 years of age) were used for cell isolation with written permission. All procedures in this study were approved by the Ethics Committee of Renmin Hospital of Wuhan University (Approval Number: WDRY-2022-K025, Wuhan, China). DPSC and A549 lung cells (iCell, Shanghai, China) were cultured in Dulbecco's modified eagle medium (DMEM) containing 10% fetal bovine serum and 1% penicillin/streptomycin, and maintained at 37 °C in a humidified atmosphere with 5% CO_2_. On reaching 80% confluency, the DPSC were characterized for MSC-associated CD markers using flow cytometry. 1 × 10^6^ DPSCs cells were incubated with antibodies against CD29 (1:25), CD44 (1:25), CD90 (1:50), CD105 (1:50), CD166 (1:50), CD14 (1:20), CD34 (1:50) and CD45 (1:25) (Abcam, Cambridge, UK) at 4 °C for 60 min. Three washes with staining buffer, the cells were suspended in 200 μl staining buffer. The flow cytometry analysis of the cells was conducted using a CytoFLEX system (Beckman Coulter, Brea, CA, USA), all experiments were repeated 3 times.

### Preparation of DPSC lysate

DPSC with 80% fusion were harvested, washed twice with phosphate buffered saline (PBS), and resuspended in DMEM (adjusted to a cell concentration to 1 × 10^9^ cells l^−1^). Subsequently, cells were lysed by repeated freeze–thaw cycles in a − 80 °C refrigerator. Following snap freezing, the lysed suspensions were maintained at 37 °C until approximately 60% of the suspension was thawed, and then transferred onto the ice until completely thawed. DPSC lysates were obtained after the above steps were repeated 3 times and stored at − 80 °C for the following experiments. For Transmission electron microscope (TEM) observation, DPSC lysate was stained by uranyl oxalate at a concentration of 2% (w/v) and observed by TEM ((HITACHI, HT7700, Japan). The nanoparticle tracking analysis (NTA) determined the size and concentration of fresh DPSC lysate using ZetaView PMX 110(Particle Metrix, Germany). Three separate batches of DPSC lysate were performed using a human cytokines array (GSH-GF-1) (RayBiotech, USA) according to the manufacturer’s instructions.

### Cell counting kit-8 (CCK-8) assay

A549 cells were seeded in 96-well plates. After adherence for 5 d, the supernatants were removed and replaced with prepared DPSC lysate at different dilutions (1:1, 1:10, and 1:20); while controls were similarly prepared by replacing the supernatant with DMEM. Subsequently, 10 μl CCK8 reagent was added to each well and incubated for 1 h at 37 °C protected from light, and the optical density (OD) was measured using a microplate reader (Biotek, Vermont, United States). Cell viability was calculated using the following formula: ${\text{cell viability}}\, = \,\left( {{\text{OD treatment}} - {\text{OD blank}}} \right)/\left( {{\text{OD control}} - {\text{OD blank}}} \right)$.

### Annexin-V and propidium iodide (PI) staining

The A549 cells treated with DPSC lysate for 5 days were harvested and washed with cold PBS. The cells were then incubated with Annexin-V-FITC and PI (Invitrogen) at room temperature for 15 min and then analyzed by flow cytometry (Beckman Coulter, Brea, CA, USA).

### Enzyme-linked immunosorbent assay (ELISA)

The A549 cells were seeded into 96-well plates at a density of 1 × 10^4^ cells per well for 24 h at 37 °C before exposed to DPSC lysate. The cells without treatment were served as a negative control. Then the carcino embryonic antigen (CEA), neuron-specific enolase (NSE), and squamous cell carcinoma antigen (SCC) level in the supernatant were determined by Human CEA ELISA kit (ab99992), Human NSE ELISA kit (ab217778) (Abcam, Cambridge, MA, USA), and Human SCC ELISA kit (YS06802B) (YaJi Biological, Shanghai, China). All procedures were performed according to the manufacturer’s instructions.

### Real-time quantitative polymerase chain reaction (qRT-PCR)

Total RNA was extracted from the A549 cells treated with DPSC lysate with Trizol reagent (Invitrogen, Carlsbad, CA, USA). The total RNA was used for complementary DNA synthesis using the TransScript All-in-One First-Strand cDNA Synthesis SuperMix for qPCR kit (Transgen, Beijing, China) as per the manufacturer’s protocol. The primers used for the reaction are listed in Table 1. The thermal conditions for the qRT-PCR were as follows- cycle 1: 95 °C for 3 min; and cycle 2 (× 45): 95 °C for 7 s, 57 °C for 10 s, and 72 °C for 15 s. The relative expression level was calculated by the 2^−ΔΔCt^ method with GAPDH as an internal reference.

**Table 1:** Specific primer sequences for qRT-PCR

Genes	Forward (5′-3′)	Reverse (5′-3′)
CEA	AGTCAGTCCCAGGCTGCAGC	GAGGACATCCAGGGTGACTG
NSE	AAGCGTGCAAGCTGGCCCAG	TGGTTGTATTTAGCCAGACG
SCC	TGGAAGAGAGCTATGACCTC	CTGCTCCCTCCTCTGTAACC
CycA	GGATGGTAGTTTTGAGTCACCAC	CACGAGGATAGCTCTCATACTGT
CycD1	GCTCGAAGTGGAAACCATC	CCTCCTTCTGCACACATTTGAA
CycE	GCCAGCCTTGGGACAATAATG	CTTGCACGTTGAGTTTGGGT
CDK2	CCAGGAGTTACTTCTATGCCTGA	TTCATCCAGGGGAGGTACAAC
Cdc25A	TTCCTCTTTTTACACCCCAGTCA	TCGGTTGTCAAGGTTTGTAGTTC
Cdc14A	CGAGCACTATGACCTCTTCTTCA	AGGCTATCAATGTCCCTGTTCT
PI3K	AGCCCAGTGACATCAACACTT	CCGCATCATAGGTCGATTTCAA
EDN1	AAGGCAACAGACCGTGAAAAT	CGACCTGGTTTGTCTTAGGTG
GRB2	ATTCCTGCGGGACATAGAACA	GGTGACATAATTGCGGGGAAAC
THBS1	GCCATCCGCACTAACTACATT	TCCGTTGTGATAGCATAGGGG
ITGB1	CAAGAGAGCTGAAGACTATCCCA	TGAAGTCCGAAGTAATCCTCCT
ITGA3	CTACCACAACGAGATGTGCAA	CCGAAGTACACAGTGTTCTGG
CLDN1	CCTCCTGGGAGTGATAGCAAT	GGCAACTAAAATAGCCAGACCT
CLDN2	GCCTCTGGATGGAATGTGCC	GCTACCGCCACTCTGTCTTTG
CSNK2B	TGAGCAGGTCCCTCACTACC	GTAGCGGGCGTGGATCAAT
F11R	GTGCCTACTCGGGCTTTTCTT	GTCACCCGGTCCTCATAGGAA
HRAS	ATGACGGAATATAAGCTGGTGGT	GGCACGTCTCCCCATCAATG
JAM3	CGGCTGCCTGACTTCTTCC	TGGGGTTCGATTGCTGGATTT
Caspase-3	TCCACAGCACCTGGTTATTA	AGGACTCAAATTCTGTTGCCA
Bcl-2	TCTTTGAGTTCGGTGGGGTCA	GTACAGTTCCACAAAGGCAT
Bax	TGCACCAAGGTGCCGGAAC	CCACAAAGATGGTCACGGTCT
Bad	TCTGGGCAGCACAGCGCTA	TGGAAGACTCGCGTCCAGC
GAPDH	GGAGCGAGATCCCTCCAAAAT	GGCTGTTGTCATACTTCTCATGG

### Transwell assay

The migration and invasion of A549 cells exposed to DPSC lysate were measured by the transwell assay. A549 cells were cultured in serum-free DMEM and embedded into DPSC lysate in the transwell chamber. After 48 h, A549 cells were fixed and stained with crystal violet, and the cells on the upper surface of the upper chamber were carefully wiped off with a clean swab. The number of A549 cells penetrating to the lower surface of the chamber was observed under a light microscope to determine the impact of the DPSC lysate on the migration ability of A549 cells. The invasion ability of A549 cells was measured by spreading Matrigel matrix gel in the upper chambers and performing the same procedure as above.

### Western blot

Tissues and cells were lysed in radioimmunoprecipitation assay buffer (Beyotime, Shanghai, China). The protein contents were determined by a BCA protein detection kit (Beyotime, Shanghai, China). The proteins were separated by electrophoresis on sodium dodecyl sulfate–polyacrylamide (SDS PAGE) gels and transferred to a polyvinylidene difluoride (PVDF) membrane. The membranes were blocked with 5% skim milk for 2 h at room temperature, and then probed with the indicated primary antibodies overnight at 4 °C with gentle shaking. After washing with phosphate buffered saline with Tween 20 (PBST), the membrane was developed with a secondary antibody (HRP-conjugated goat anti-rabbit IgG; ab205718) for 1 h at room temperature. Subsequently, membranes were visualized using a chemiluminescence imaging system (Biolight, Guangzhou, Guangdong, China) according to the manufacturer’s protocol. The primary antibodies used were anti-CycA (ab185619, 1:1000), anti-CycD1 (ab16663, 1:100), anti-EDN1 (ab2786, 1:1000), anti-PI3K (ab278545, 1:1000), anti-CLDN1 (ab180158, 1:2000), anti-CLDN2 (ab53032, 1:500), anti-Caspase-3 (ab184787, 1:2000), anti-Bcl-2 (ab182858, 1:2000), anti-Bax (ab32503, 1:1000), anti-Bad (ab32445, 1:1000), anti-GAPDH (ab8245, 1:500) from Abcam (Cambridge, MA, USA).

### Animal experiments

Male nude mice aged 6–8 weeks were obtained from SPF (Beijing) Biotechnology Co., Ltd (Beijing, China). Mice were kept at 26 °C ± 2 ℃ and 40–60% humidity with a 12 h light/dark cycle. The animals were housed in individual cages and fed with free access to food and water. This study was approved by the Experimental Animal Ethics Committee of Zhejiang Haikang Biological Products Co. Ltd. (HKSYDWLL2021021) and followed the Guide for the Care and Use of Laboratory Animals, published by the US National Institutes of Health. Twelve mice were subcutaneously injected with 1 × 10^6^ A549 cells and waited for a week until the tumors were seen, then divided into four groups: control group, DPSC lysate 1:1 group, DPSC lysate 1:10 group, and DPSC lysate 1:20 group. Each of the DPSC lysate groups were injected intraperitoneally with the corresponding dilution of the lysate (25 ml kg^−1^) once per day for 14 days, and the control group was given an equal amount of saline. The animals were monitored for tumors for 4 weeks, and tumor size was recorded every 7 days. The mice were injected with sodium pentobarbital and sacrificed on day 28. The tumors were resected, weighed, and then stored at − 80 °C. The formula for tumor volume (V) is V = 0.5 × a × b^2^, where a is the longest diameter and b is the shortest diameter.

### Statistical analysis

Data are represented as means ± SD. Prism 8.0 (Graphpad Software, California, USA) was utilized for statistical analyses. For comparisons among multiple groups, one-way ANOVA was used if it conformed to a normal distribution, otherwise the Friedman test was used. *p* < 0.05 was considered significant difference.

## Results

### Characterization of DPSC

**Fig. 1:**
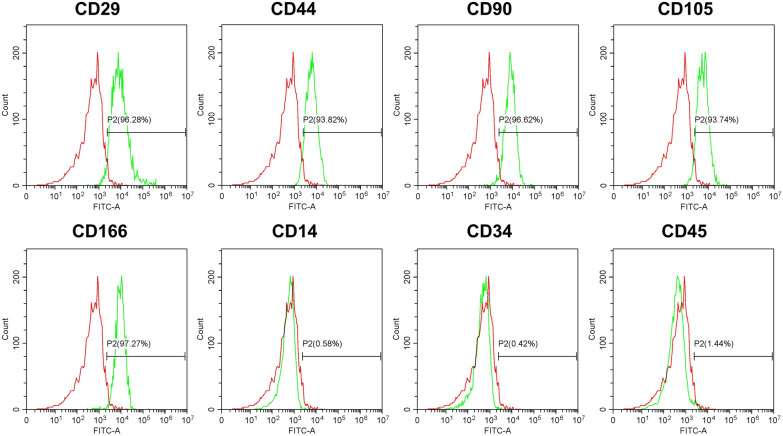
Characterization of DPSC. Immunophenotyping of DPSC using flow cytometry analysis. The percentage of positively stained cells is indicated in the middle right section of each histogram. The green line indicates the positively stained cells; whereas the red line indicates the isotype-matched monoclonal antibody control. Histograms showed that DPSC were positive for CD29, CD44, CD90, CD105, CD166, and were negative for CD14, CD34, CD45

First, DPSC were identified by analyzing the surface markers on the cells. Flow cytometry analysis showed a high expression of CD29 (96.28%), CD44 (93.82%), CD90 (96.62%), CD105 (93.74%), CD166 (97.27%); and a lack of expression of CD14 (0.58%), CD34 (0.42%), and CD45 (1.44%) (Fig. 1). This pattern of expression of cell surface markers is associated with mesenchymal stem cells [30].

### Characterization of DPSC lysate

**Fig. 2:**
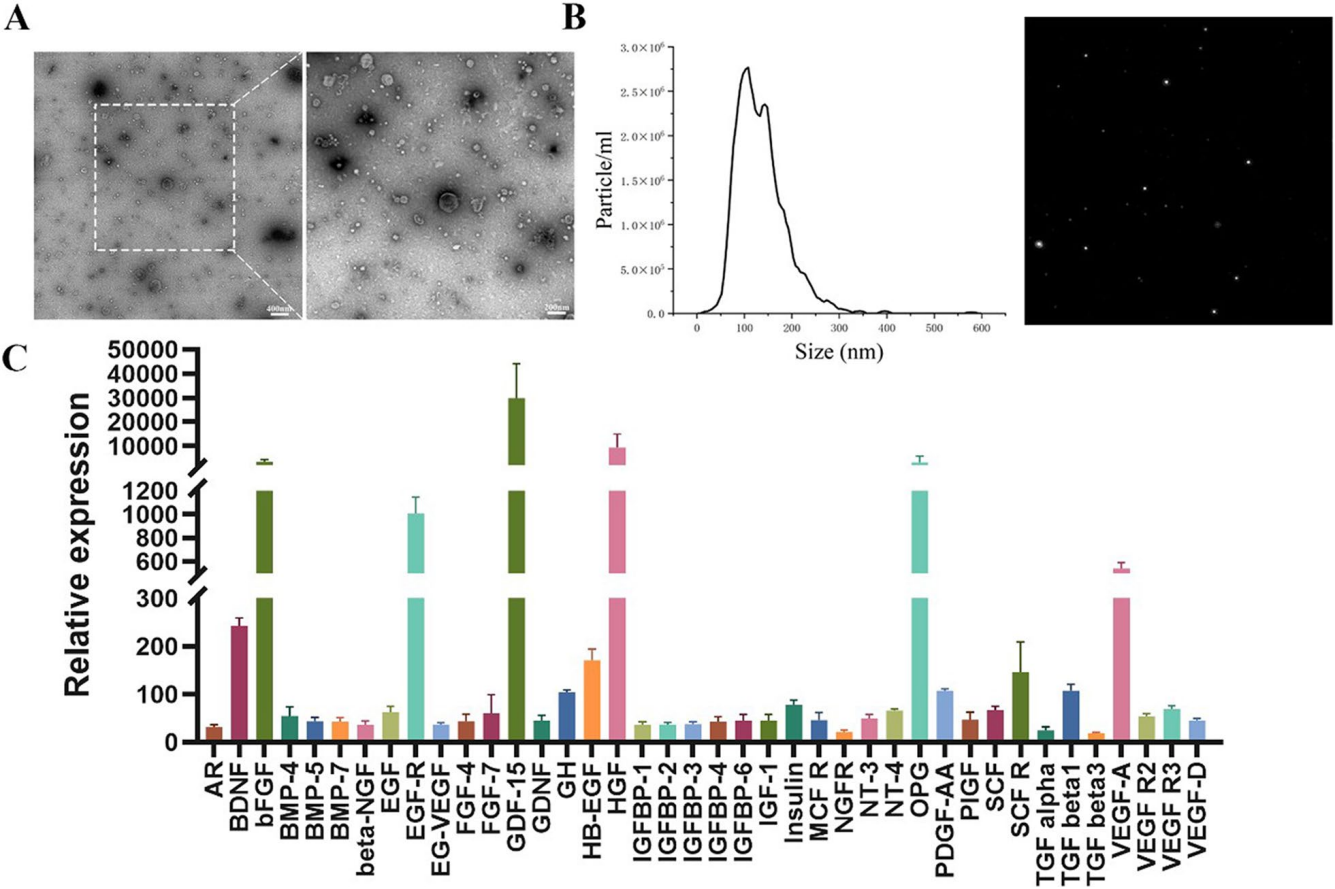
Characterization of DPSC lysate. **A** TEM photograph of DPSC lysate with negative staining, scale bar: 400 nm or 200 nm. **B** Size distribution measurement of DPSC lysate by NTA analysis, and NTA video image of DPSC lysate under Brown motion. **C** Cytokine array of DPSC lysate by densitometric analysis (n = 3)

DPSC lysate contains abundant vesicles, many cup-shaped morphology of vesicles can be observed by TEM (Fig. 2A). As shown in Fig. 2B, the size distribution and concentration of DPSC lysate were detected by using NTA. The exosomes ranged from 37 to 343 nm in diameter with a peak at 108 nm. These results were in good agreement with the previous reports on DPSC-derived exosome [31]. DPSC lysate is rich in cell growth factors. Cytokine arrays (Fig. 2A) showed that all 40 kinds of cell growth were expressed and the significantly enhanced protein in DPSC lysate was bFGF, EGF-R, GDF-15, HGF, OPG and VEGF-A.

### DPSC lysate inhibited A549 cells proliferation and promoted apoptosis

**Fig. 3:**
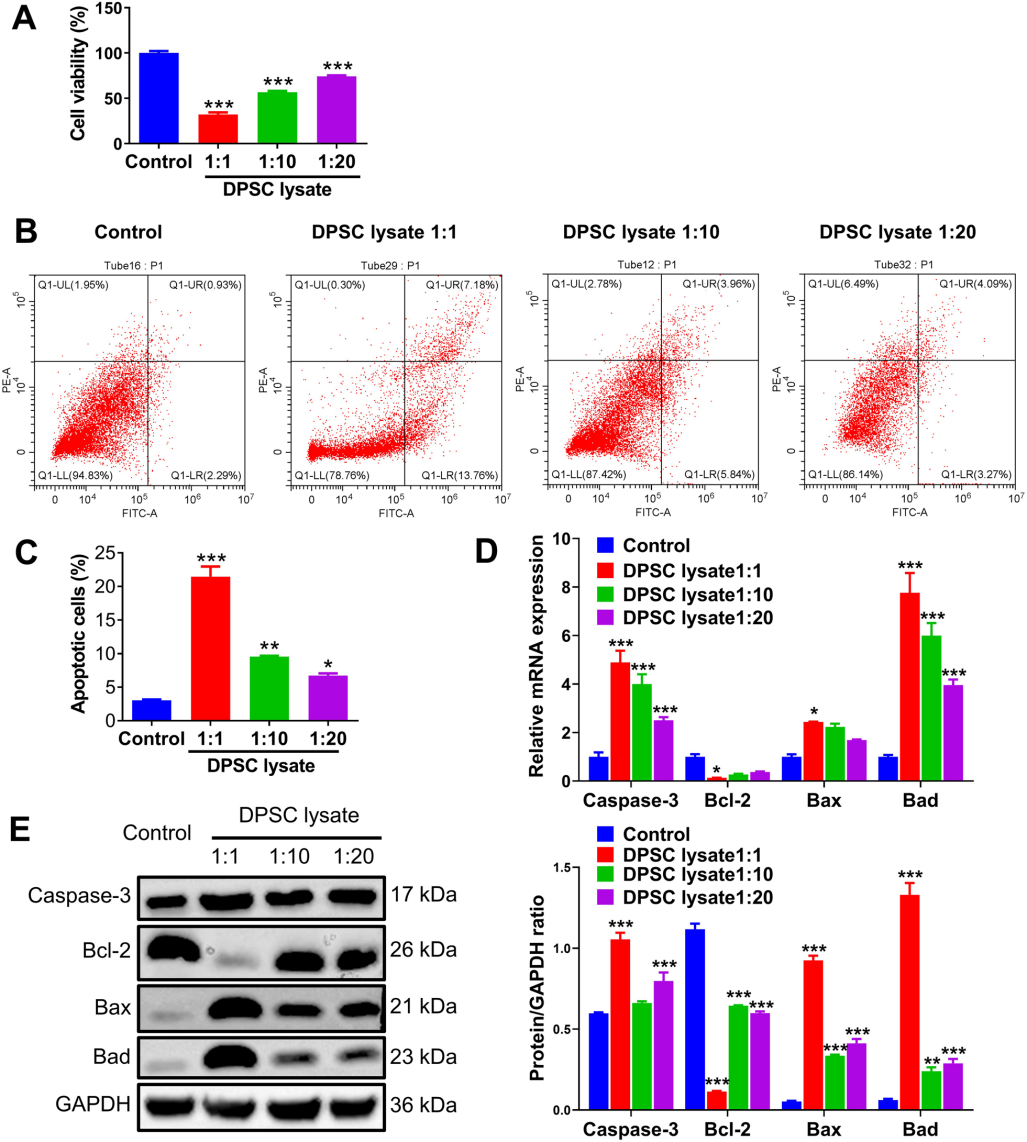
DPSC lysate inhibits A549 cells proliferation and promotes apoptosis. **A** CCK-8 was used to detect the effect of DPSC lysate on the viability of A549 cells. **B** Flow cytometry was used to detect the effect of DPSC lysate on the apoptosis of A549 cells (n = 3). **C** The histogram was used to reflect the percentage of apoptotic cells. **D** The expression of Caspase-3, Bcl-2, Bax, and Bad was detected by qRT-PCR (n = 3). **E** The level of Caspase-3, Bcl-2, Bax, and Bad was detected by western blot,total proteins were normalized to the intensity of GAPDH(n = 3). *p < 0.05, **p < 0.01, ***p < 0.001

The inhibitory effect of DPSC lysate on the viability of A549 lung cancer cells was assessed using CCK-8. DPSC lysate decreased the proliferation of A549 cells compared to the control group (*p* < 0.001, Fig. 3A). In addition, apoptosis was detected by Annexin-V and PI staining. It is evident from Fig. 3B and C that DPSC lysate promoted apoptosis in A549 cells. Meanwhile, the role of DPSC lysate in A549 cells apoptosis was verified by qRT-PCR and western blot, and DPSC lysate was found to upregulate the expression of Caspase-3, Bax, and Bad, and downregulate the expression of Bcl-2 (Fig. 3D and E).

### DPSC lysate inhibited the migration and invasion of A549 cells

**Fig. 4:**
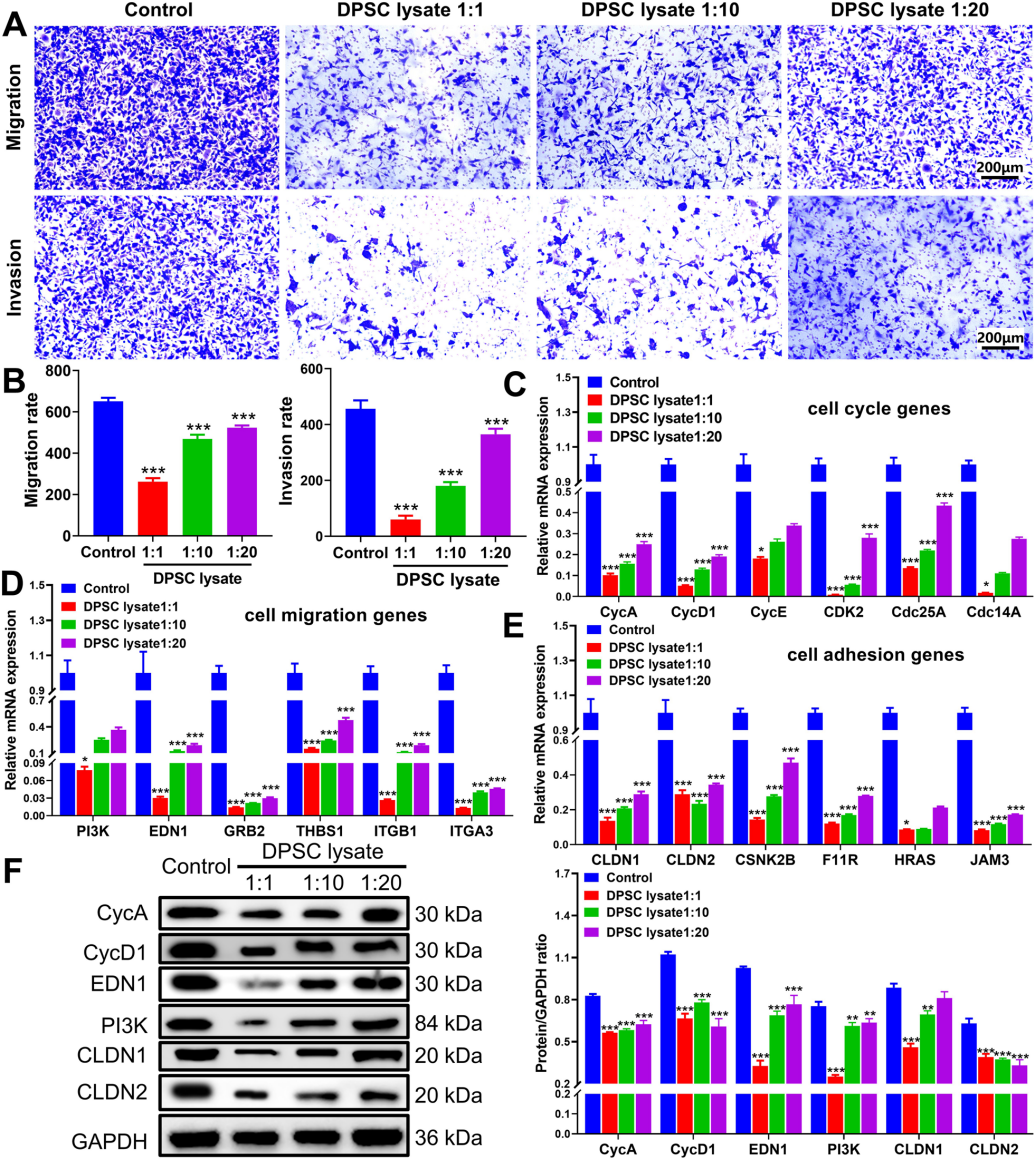
DPSC lysate inhibited the migration and invasion of A549 cells. **A** Transwell assay was used to detect the effect of DPSC lysate on the migration and invasion of A549 cells (n = 3). Scale: 200 μm; magnification: 100 ×. **B** The histogram was used to reflect the migration and invasion rate. The key genes about **C** cell cycle, **D** cell migration and **E** cell adhesion were detected by qRT-PCR (n = 3). **F** The key genes about cell cycle (CycA and CycD1), cell migration (EDN1 and PI3K) and cell adhesion (CLDN1 and CLDN2) were detected by western blot,total proteins were normalized to the intensity of GAPDH (n = 3). *p < 0.05, **p < 0.01, ***p < 0.001

To examine the migratory and invasive properties of A549 cells, we conducted the transwell assay. The results showed that the DPSC lysate could reduce the migration and invasion of A549 cells as compared to the control group (Fig. 4A and B). qRT-PCR and western blot were performed to detect key genes for cell cycle, migration, and adhesion. At the level of mRNA, some key genes involved in the cell cycle (*CycA*, *CycD1*, *CycE*, *CDK2*, *Cdc25A*, and *Cdc14A*), cell migration (*PI3K*, *EDN1*, *GRB2*, *THBS1*, *ITGB1*, and *ITGA3*), and cell adhesion (*CLDN1*, *CLDN2*, *CSNK2B*, *F11R*, *HRAS*, and *JAM3*) were downregulated in DPSC lysate, compared with the control group (*p* < 0.001, Fig. 4C–E). The changes in the protein levels of CycA, CycD1, EDN1, PI3K1, CLDN1, and CLDN2 were further detected with western blot. The results were consistent with qPCR and showed that the expression of these genes was upregulated in the groups of DPSC lysate, compared with the control group (*p* < 0.001, Fig. 4F).

### DPSC lysate downregulated CEA, SCC, NSE in supernatant of A549 cells

**Fig. 5:**
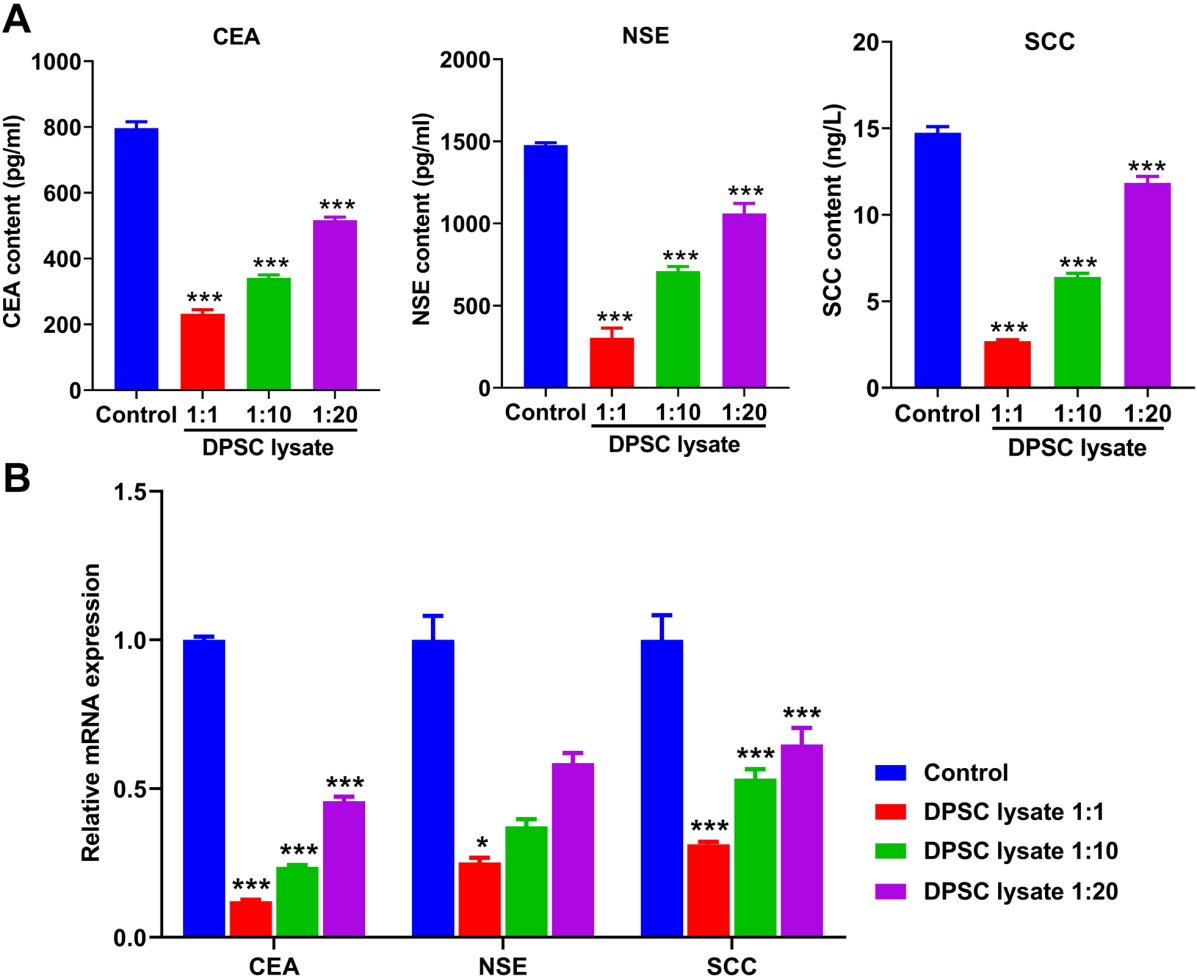
DPSC lysate downregulates content of CEA, SCC, NSE in supernatant of A549 cells. **A** The protein level of CEA, NSE, and SCC was detected by ELISA (n = 3). **B** The mRNA expression of CEA, NSE, and SCC was detected by qRT-PCR (n = 3). *p < 0.05, ***p < 0.001.

CEA, SCC, and NSE are markers of lung cancer. Using ELISA and qRT-PCR assays, we found that there were decreased levels of CEA, SCC, and NSE in the supernatant of A549 cells treated with DPSC lysate (*p* < 0.001, Fig. 5).

### DPSC lysate inhibited tumor growth in vivo

**Fig. 6:**
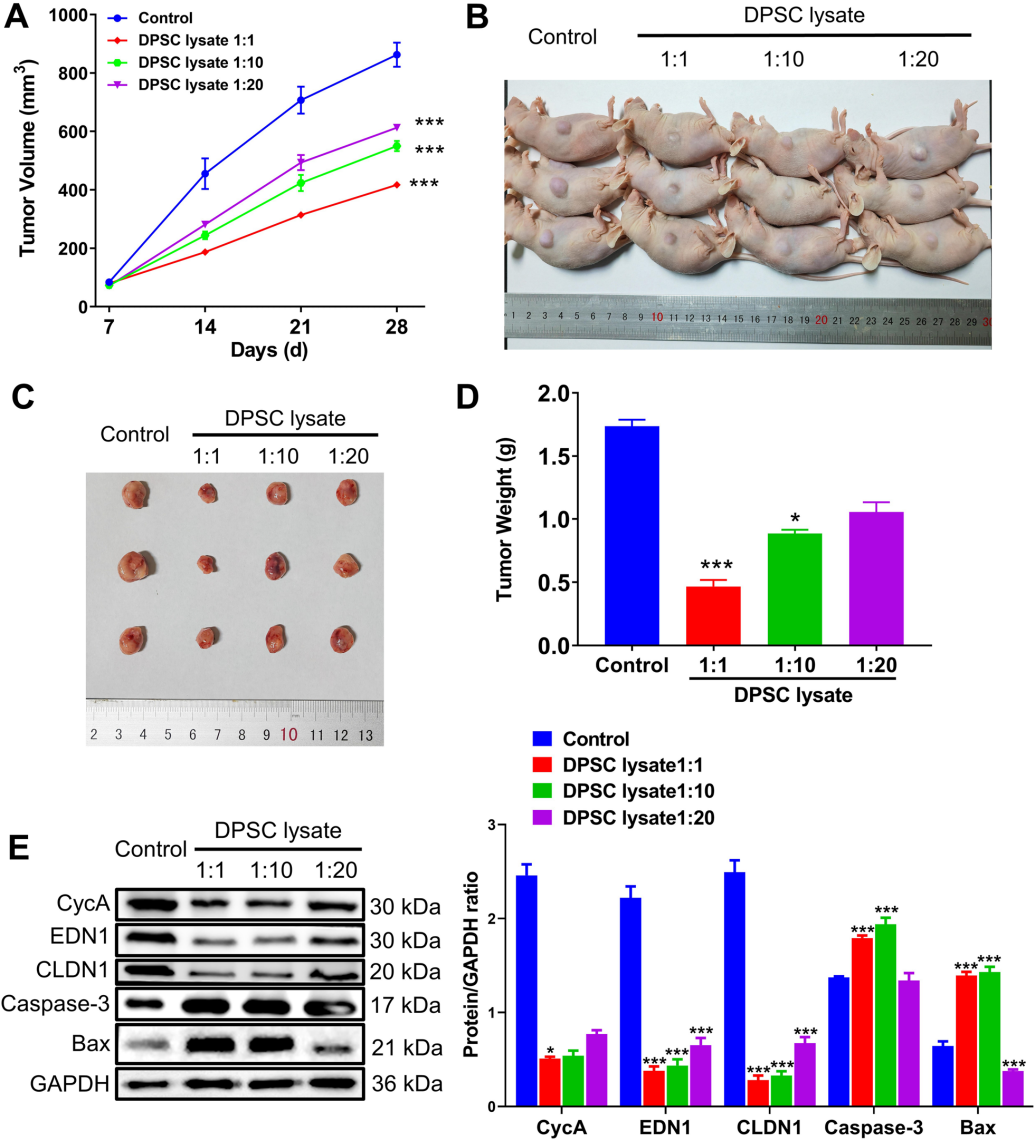
DPSC lysate inhibits tumor growth in vivo. **A** Tumor growth curves of tumor volume after different treatments for 28 days. **B** tumor-bearing mice with different treatments. **C** and **D** Tumor images and weight of mice with different treatments after 28 days. **E** Western blot of CycA, EDN1, CLDN1, Caspase-3, and Bax. (n = 3) *p < 0.05, ***p < 0.001

DPSC lysate was injected intraperitoneally into nude mice tumor model to observe the inhibitory effect of DPSC lysate on tumors in vivo (Fig. 6A). After 28 days of DPSC lysate treatment, both tumor volume and tumor weight were significantly inhibited compared to the control group (*p* < 0.001, Fig. 6B–D). Subsequently, protein levels of CycA, EDN1, CLDN1, and pro-apoptotic proteins (Caspase-3 and Bax) in tumor tissues were detected by western blot. Compared with the control group, the protein levels of CycA, EDN1, and CLDN1 were significantly upregulated, while the apoptotic gene was significantly upregulated (*p* < 0.001, Fig. 6E).

## Discussion

DPSC, a type of MSCs derived from dental pulp, have been shown to have therapeutic effects in many diseases [32–35]. However, there is much controversy about the role of MSCs in cancer. DPSC promoted the proliferation and metastasis of PC-3 cancer cells under in vitro co-culture conditions [36]. However, DPSC-conditioned medium had significant apoptotic and growth inhibitory effects on CRC cells through the MAPKinase and apoptotic signaling pathways [37]; and human MSCs derived from the placenta and the chorionic villus inhibited proliferation and enhanced migration of human breast cancer cells [38]. MSCs may have two conflicting effects on different types of tumor cells, either promoting or inhibiting them. On the one hand, MSCs provide a framework for fixing tumor cells in the form of a tumor stroma and secrete factors that promote tumor growth. On the other hand, MSCs present in the tumor microenvironment can transdifferentiate into M2 macrophages or bone marrow-derived suppressor cells under the influence of cytokines or chemokines to suppress tumors [39]. The effects of DPSC on lung cancer are unknown so far.

In our study, we investigated the role of DPSC lysate in lung cancer using the A549 cell line. Interestingly, DPSC lysate was able to effectively reduce the viability of lung cancer cells and induce apoptosis in a concentration-dependent manner. Western blot results showed that the expression levels of pro-apoptotic proteins in the 1:1 group of DPSC lysate were much higher than those in the other two lysate groups, probably because the high concentration of 1:1 DPSC lysate caused changes in the osmolarity of the cancer cell environment, leading to apoptosis [40]. Apoptosis is a programmed death and is an important self-stabilizing mechanism in organisms. Bcl-2 proto-oncogene is located in the mitochondrial membrane, and high expression of Bcl-2 can inhibit apoptosis. Bax is mainly located in the cytoplasm and mediates the release of downstream apoptotic molecules, thus triggering apoptosis. The downregulation of Bax/Bcl-2 ratio is common in various tumors and is a key determinant of apoptosis [41, 42]. Caspase-3 is an important protein in the apoptotic pathway, and several apoptotic pathways induce apoptosis through Caspase-3 signaling [43].

Western blot results showed that Caspase-3, Bax and Bad protein levels increased and Bcl-2 protein levels decreased after treatment of A549 cells with DPSC lysate for 3 days. These results suggest that DPSC lysate inhibits A549 cell proliferation and induces apoptosis through an intrinsic mitochondria-mediated pathway. In addition, we found that DPSC lysate reduced the migration and invasion of cancer cells and significantly downregulated the expression of mRNAs for a series of cell cycle-, cell migration-, and cell adhesion-related genes. The ELISA and qRT-PCR assays revealed that the mRNA expression of tumor markers (NSE SCC CEA) as well as protein expression in tumor cells were significantly decreased after treatment with DPSC. Finally, when we studied whether the DPSC lysate inhibited in vivo tumor growth in mice, the results were the same as the cellular assay, further validating the inhibitory effect of DPSC lysate on A549 cells. The DPSC lysate inhibited tumor both in vivo and in vitro. Interestingly, Cytokine arrays showed that significantly enhanced protein in DPSC lysate was bFGF, EGF-R, GDF-15, HGF, OPG and VEGF-A, which all aids in the expansionary of tumors. This contradictory result suggests that the inhibitory effect of DPSC on tumor is not achieved by cytokines, and this inhibitory effect is much stronger than the pro-tumor effect of cytokines. Bone marrow mesenchymal stem cell (BMSC) and umbilical cord mesenchymal stem cell (UMSC) derived EVs also showed inhibitory effects on different tumors. BMSC derived-evs could suppress lung cancer via KDM3A/DCLK1/FXYD3 axis [44]. The anti-proliferative and pro-apoptotic effects of EVSs derived from UMSC were detected in bladder carcinoma [45].There are some reports suggested that IL-1β blockade may be a preventive strategy for high risk individuals [46]. In our previous study, we found that both DPSC and DPSC lysate could significantly inhibit the expression of IL-1β [19, 47]. This may be one of the mechanisms by which DPSC lysate inhibits tumor. DPSC lysate shows an inhibitory effect on lung cancer cells, and has a higher acquisition rate, easier to standardize. Compared with exosomes, it is more suitable for clinical application. This study has some limitations. The first limitation is that in vitro experiments were conducted only on the A549 cell line, whereas multiple lung cancer cell lines need to be selected in future studies. Secondly, further experimentation is needed to elucidate a more detailed mechanism, as evidenced by the current lack of sufficient references.

## Conclusion

In summary, DPSC lysate inhibits A549 cell proliferation and migration, promotes endogenous pathway apoptosis, and downregulates some genes associated with the cell cycle, migration, and adhesion. Thus, DPSC lysate is expected to provide a cell-based alternative therapy for lung cancer treatment.

## Data Availability

The data that support the findings of this study are available from the corresponding authors upon reasonable.
